# Assessing the psychometric properties of the 10-item version of the comprehensive assessment of ACT processes (CompACT) in a community-based adult sample

**DOI:** 10.1016/j.jbct.2025.100569

**Published:** 2025-12-24

**Authors:** Michael Christopher, Marissa Ferry, Olivia Boyd, Xander Kahle, Salena Keys-Kukoricza, J. Morgan Penberthy, Kaylia Pham

**Affiliations:** a College of Health Sciences, Boise State University, 1910 University Drive, Boise, ID 83725 USA; b School of Graduate Psychology, Pacific University, USA; c Graduate School of Education and Counseling, Lewis & Clark College, USA

**Keywords:** Psychological flexibility, Confirmatory factor analysis, Acceptance and commitment therapy

## Abstract

**Background:**

The Comprehensive Assessment of ACT Processes (CompACT) is a 23-item self-report measure of psychological flexibility with evidence of a three-factor structure, convergent and discriminant validity, and reliability. Several short-form CompACT versions, including 18-, 15-, and 10-item forms have been developed. The primary aim of this study was to compare the model fit of each short form and to further examine the convergent and discriminant validity and reliability of the best fitting model in an adult community-based sample in the United States.

**Method:**

Participants were recruited for the study via a Qualtrics participant panel. A total of 601 participants completed the CompACT and other self-report measures.

**Results:**

A series of confirmatory factor analyses indicated the 10-item version provided the best fit to the data. The 10-item CompACT demonstrated adequate reliability and evidence of convergent and discriminant validity with correlations in the expected direction with psychological distress, life satisfaction, mindfulness, and resilience, but not gender.

**Discussion:**

Despite limitations, these results suggest the 10-item CompACT is a valid and reliable measure of psychological flexibility.

## Introduction

Psychological flexibility, or the ability to contact the present moment more fully as a conscious human being, and to change or persist in values-aligned behavior, is a core element of Acceptance and Commitment Therapy (ACT; Hayes et al., 2003). The most thoroughly studied measure of psychological flexibility is the Acceptance and Action Questionnaire – II (AAQ-II; (Bond et al., 2011). Despite evidence of good psychometrics, AAQ-II limitations include that it is unidimensional and may lack discriminant validity (Tyndall et al., 2019; Wolgast, 2014). The Comprehensive Assessment of ACT Processes (CompACT; Francis et al., 2016) is a 23-item self-report measure designed in response to perceived shortcomings of the AAQ-II. Through a two-step process including a Delphi-consensus poll and psychometric testing using a non-clinical United Kingdom (U.K.) sample, the initial 106-item pool was reduced to a 23-item measure. Consistent with expectations, the 23 items fit a three-factor solution and had a Cronbach’s alpha of 0.91. The CompACT is composed of three factors: openness to experience (OE; eight acceptance-related items and two defusion-related items), behavioral awareness (BA; five present moment awareness-related items), and valued action (VA; eight values/committed action-related items). A subsequent CompACT confirmatory factor analysis (CFA) replicated the three-factor correlated structure (Bayliss, 2018).

The CompACT has recently been translated into French (Spencer et al., 2025), Italian, German, and Spanish (Giovannetti et al., 2022; Giovannetti et al., 2024), Czech (Ptacek & Jelínek, 2024), Malay (Musa et al., 2022), Chinese (Chen et al., 2020), Persian (Soleimani & Shairi, 2021), Portuguese (Trindade et al., 2021), and Romanian (Calinici & Calinici, 2021). Results from these studies indicate evidence of reliability and validity of the 23-item CompACT ranges from poor to acceptable, and some versions deleted items to improve model fit. Variability in the CompACT’s psychometric performance across studies appears to be attributable to several factors, including differences in sample characteristics (i.e., clinical vs non-clinical) and cultural variations in the meaning of items used to measure psychological flexibility. For example, in developing the Chinese version of the CompACT, Chen et al. (2020, p. 15530) identified potentially problematic items based on expert feedback regarding “feelings”, “thoughts”, and the concept of “importance” because all are for specific things or present states, and the items may have been difficult for participants to understand and easily confused with the meaning of other items.

In addition to translated and culturally adapted versions, several researchers recently developed CompACT short forms, including 18-item (Trindade et al., 2021; Tynan et al., 2022), 15-item (Hsu et al., 2023), and 10-item (Golijani-Moghaddam et al., 2023) forms. In examining the CompACT using an EFA, Trinidade et al. (2021) identified 5 items that cross loaded (items 6, 13, 18, 20, 22) which were removed, and the subsequent 18-item version provided an adequate fit to the data. In a follow-up study, the 18-item version provided a better fit to the data than the 23-item version among a U.S. military sample (Tynan et al., 2022). Hsu et al. (2023) similarly found the 23-item CompACT provided a poor fit to the data. They removed eight items with redundant content, inconsistent wording direction within a factor, or that required familiarity with ACT concepts (items 5, 6, 13, 14, 18, 20, 21, 22). The resulting 15-item CompACT provided a good fit to the data, a stable 3-factor structure, and evidence of measurement consistency over time. Most recently, several developers of the 23-item CompACT (Francis et al., 2016) set an *a priori* goal of maximizing brevity while retaining reliability, stability, and theoretical scope in creating a short-form CompACT measure by removing 50 % of items from each subscale, only retaining those with the highest factor loadings, resulting in a 12-item measure (items 2, 3, 5, 8, 10, 11, 12, 14, 15, 19, 22, 23 were removed; Golijani-Moghaddam et al., 2023). In a large adult U.K. sample, a CFA of the 12-item CompACT provided an adequate fit to the data; however, two additional items (2, 22) had factor loadings below a set cutoff (0.60) and were removed. The resulting 10-item CompACT provided an excellent fit to the data, demonstrated evidence of reliability, and correlated in the expected direction with related constructs (Golijani-Moghaddam et al., 2023). Although the U.K. shares many similarities with U.S., subtle differences in culture and language expressions necessitates examination of the 10-item CompACT psychometrics in the U.S., as well as other cultures and languages. Moreover, although evidence supports the psychometrics of the 18-, 15-, and 10-item versions of the CompACT, the question remains as to which provides the best and most parsimonious fit to the data. Therefore, the primary aim of this study was to determine the best fitting CompACT model, and to explore its factorial, convergent, discriminant validity and reliability in a community-based U.S. adult sample.

## Method

### Participants and procedure

Following Pacific University IRB approval, participants were recruited through Qualtrics’ online panel service. A Qualtrics panel is a group of participants who are recruited using targeted criteria (https://www.qualtrics.com/support/survey-platform/distributions-module/online-panels/). The criteria for this study (which also served as the inclusion criteria) included adults age 18–70 living in the U.S., fluency in English, access to the internet for the survey, and completion of a high school education or equivalent. Participants completed self-report measures online via Qualtrics and were offered an incentive for their time (a $4 credit toward a gift card). Participants provided informed consent and were ensured their participation was voluntary and their responses were confidential. A total of 749 participants consented to participation and 727 provided basic demographic information. Of these, 601 completed all study measures without any missing data and formed the final study sample (i.e., complete case analysis), and all 601 participants passed four attention checks (e.g., please select the option “seldom”). Completers were compared to non-completers on available data (using *t* or chi-square tests) and there were no statistically significant differences between the groups (all *p*’s > 0.10). An *a priori* power calculation with an estimated medium effect size (*d* = 0.30), power of 0.8 and three latent variables (BA, OE, VA) indicated a minimum of 256 participants were needed to detect an effect and for model convergence (Soper, 2021 [https://www.danielsoper.com/statcalc/default.aspx]). We purposely oversampled to help ensure model convergence.

### Measures

The CompACT (Francis et al., 2016) is a 23-item measure of psychological flexibility (psychometrics described above). Items are scored on a 7-point Likert scale ranging from 0 = *strongly disagree* to 6 = *strongly agree*. Higher scores indicate greater psychological flexibility (measure psychometrics are described above).

The Satisfaction with Life Scale (SWLS; Diener et al., 1985) is a 5-item measure of global life satisfaction. Items are rated on a 7-point Likert scale ranging from 1 = *strongly disagree* to 7 = *strongly agree*. Total scores range from 5 to 35 with higher scores indicating higher satisfaction with life. The SWLS has demonstrated good internal consistency (α = 0.87), test–retest reliability (*r* = 0.82), and expected correlations with psychological distress (Diener et al., 1985) and psychological flexibility (Marshall & Brockman, 2016).

The Brief Resilience Scale (BRS; Smith et al., 2008) is a 6-item measure used to assess psychological resilience. Specifically, the BRS examines the ability to recover or “bounce back” from stressors. Items are rated on a 5-point Likert scale ranging from 1 = *strongly disagree* to 5 = *strongly agree*. A total score is calculated by averaging responses to the 6 items, resulting in a range of 1 to 5 with higher scores indicating greater psychological resilience. The BRS demonstrated good internal consistency (α = 0.80) and expected correlations with psychological distress, wellbeing (Smith et al., 2008), and psychological flexibility (Christopher et al., 2018).

The Five Facet Mindfulness Questionnaire-Short Form (FFMQ-SF; Bohlmeijer et al., 2011), a 24-item version of the FFMQ (Baer et al., 2006), assesses the dispositional tendency to be mindful in daily life. Items are rated on a 5-point Likert-type scale ranging from 1 = *never or very rarely true* to 5 = *very often or always true*. The observe and describe facets of the scale have demonstrated weaker psychometric properties and issues with novice and non-meditating samples (De Bruin et al., 2012; Lilja et al., 2013). Thus, the current study used three of the five facets—acting with awareness, nonjudging of experience, and nonreactivity to inner experience to create a total mindfulness score. Each facet has five items, resulting in a 15-item scale that ranges from 15 to 75, with higher scores indicating greater mindfulness. The FFMQ-SF demonstrated good internal consistency (α = 0.82), factorial validity, and expected correlations with psychological flexibility, wellbeing, and psychological distress (Bohlmeijer et al., 2011).

The Self-Compassion Scale-Short Form (SCS-SF; Raes et al., 2011) is a 12-item version of the 26-item SCS (Neff, 2003). It assesses kindness and understanding toward oneself in instances of pain or failure, perception of one’s experiences as part of the larger human experience, and the ability to hold painful thoughts and feelings in mindful awareness. Items are rated on a 5-point Likert-type scale ranging from 1 = *almost never* to 5 = *almost always*. The SCS-SF ranges from 12 to 60, with higher scores indicating greater self-compassion. The SCS-SF demonstrated good internal consistency (α = 0.87) and expected correlations with psychological flexibility, wellbeing, and psychological distress (Macbeth & Gumley, 2012).

The Depression, Anxiety, and Stress Scale-21 (DASS-21; Henry & Crawford, 2005), a 21-item version of the DASS (Lovibond & Lovibond, 1995), is a three-factor measure of cognitive and physical experiences of negative emotional states (depression, anxiety, and stress). Each factor consists of seven items rated on a 4-point Likert-type scale ranging from 0 = *did not apply to me at all* to 3 = *applied to me very much or most of the time*. Factor scores are summed and multiplied by two, resulting in a factor score range from 0 to 42 with higher scores indicating greater distress. The DASS-21 factors have demonstrated evidence of good to excellent internal consistency (depression α = 0.88, anxiety α = 0.82, and stress α = 0.90), factorial validity (Henry & Crawford, 2005), and expected correlations with psychological flexibility (Tyndall et al., 2019).

### Study design and analytic plan

A single time point cross-sectional data collection study design was used. To assess factorial validity, three-factor (OE, VA, BA) item-level CompACT CFA models were tested using robust diagonal weighted least squares (RDWLS) estimation with LISREL Academic (Jöreskog & Sörbom, 2025). RDWLS was used due to the ordinal scale nature of the CompACT (i.e., Likert items) and related issues of multivariate nonnormality. A procedure described by (Jöreskog, 2004) was used in which polychoric correlations were estimated and then rescaled into a polychoric covariance matrix. Four competing CompACT three-factor correlated models were tested: 1) 23-item, 2) 18-item, 3) 15-item, and 4) 10-item. All participants completed the 23-item CompACT, and data from each participant were reused in the 18-, 15-, and 10-item CFAs. To assess the fit of the data to the models, four fit indices were evaluated: the Satorra-Bentler adjusted chi-square χ^2^ (SBχ^2^; Satorra & Bentler, 2001), the comparative fit index (CFI; Bentler, 1990), the root mean square error of approximation (RMSEA; Browne et al., 1993), and the standardized root mean squared residual (SRMR; Hu & Bentler, 1999). RMSEA values of 0.06 or less are thought to indicate a close fit, 0.08 a fair fit, and 0.10 a marginal fit. CFI values of 0.90 and greater, and SRMR values of approximately 0.09 or less, tend to indicate good fit (Hu & Bentler, 1999). Because the four CFA models were non-nested, the Bayesian Information Criteria (BIC; Schwarz, 1978) was used to compare model fit and parsimony, with lower values indicating a better fit. Cronbach’s alpha was used to assess internal consistency and Pearson’s correlations to assess convergent and discriminant validity. We expected the CompACT to be positively correlated with the SWLS, BRS, FFMQ-SF, SCS-SF, negatively correlated with the DASS-21 (depression, anxiety, and stress), and uncorrelated with gender.

## Results

The final sample was 53 % female (317 = female, 284 = male) and ranged in age from 18-70 years old (*M* = 41.71, *SD* = 13.01). In terms of race and ethnicity, 81.5 % of the sample identified as White American, 11 % as Black/African American, 4 % as Asian American, 1.5 % as other, 1.3 % as multiracial, and 0.7 % as Native American. Approximately 8 % of the sample identified as Latino/a. Years of education completed ranged from 12 to 35 (*M* = 14.74, *SD* = 2.92) and 44 % (*n* = 268) were married, 35 % (*n* = 203) single, 11 % (*n* = 63) divorced, 8 % (*n* = 48) cohabitating, 2 % (*n* = 11) widowed, and 1 % (*n* = 7) other.

As shown in Table 1 fit of the three-factor correlated 10-item CompACT model provided the best fit to the data: $\text{SB}{\text{χ}}_{\left(20.70\right)}^{2}=81.96$, *p* < 0.001; CFI = 0.97, RMSEA = 0.07 (90 % confidence interval = 0.060.08), SRMR = 0.06, BIC = 14,179.43. A comparison of BIC values indicated that the 10-item model provided the most parsimonious fit to the data relative to the 23–, 18-, and 15-item models. All items significantly loaded onto their respective latent factor, except for items 13, 20, and 22 in the 23-item version (see Table 2). The total CompACT-10 and each factor demonstrated acceptable to good reliability (CompACT-10 α = 0.74, BA α = 0.84, OE α =0.74, VA α = 0.82), and as shown in Table 3, CompACT-10 and its factors significantly correlated in the expected direction with measures of satisfaction with life, psychological resilience, dispositional mindfulness, self-compassion, and psychological distress (except for VA and depression/anxiety), and were unrelated to gender.

## Discussion

The primary aim of this study was to determine the best fitting CompACT model (23–, 18-, 15-, or 10-item) and to explore its factorial, convergent, discriminant validity and reliability in a community-based U.S. adult sample. The current sample differed from the original CompACT validation study (Francis et al., 2016) in nationality (i.e., U.S. versus U.K.), gender (53 % identified as female versus 74 %), and race/ethnicity (82 % identified as White, 11 % Black, and 8 % Latino/a versus 94 % White). Results from the current CFA replicated the three-factor correlated structure reported in the full 23-item (Francis et al., 2016), 18-item (Trindade et al., 2021; Tynan et al., 2022), 15-item (Hsu et al., 2023), and 10-item (Golijani-Moghaddam et al., 2023) models.

Results from the present study demonstrated a similar model fit, factor loadings, internal consistency, and correlations with other constructs as the 18-item (Trindade et al., 2021; Tynan et al., 2022), 15-item (Hsu et al., 2023), and 10-item validation studies (Golijani-Moghaddam et al., 2023). The 18-, 15-, and 10-item versions demonstrated a good fit to the data with equivalent RMSEA values, similar CFI and SRMR values (although slightly less ideal in the 18- and 15-item versions), and statistically significant factor loadings. Because these competing models were non-nested, BIC was used to assess model fit and parsimony, and the 10-item version provided the best model fit. Of note, the 23-item version provided a poor fit to the data, and three OE items were not statistically significant , which replicated Trindade et al.’s (2021) finding in developing the 18-item version. Total CompACT-18, –15, and –10 scores and the three factors demonstrated acceptable internal consistency with an observed range similar to findings reported in each of the short-form validation studies. Similar to the CompACT-18, –15, and –10 validation studies, total CompACT-10 scores and most of the three factors were significantly correlated in the expected direction with psychological distress (Golijani-Moghaddam et al., 2023; Hsu et al., 2023; Tynan et al., 2022), dispositional mindfulness (Trindade et al., 2021), psychological resilience (Hsu et al., 2023), and satisfaction with life (Tynan et al., 2022). These results provide additional support for the CompACT-10 as a useful and efficient measurement of psychological flexibility (and its subfactors) in a U.S. population.

An unexpected finding in the current study is that VA was uncorrelated with stress and anxiety. The VA factor on the CompACT-10 reflects an individual’s ability to take committed actions that align with identified values, even when it is challenging. In the absence of committed action, consequences such as narrowing of behavior via inaction and passivity or excessive unhelpful behaviors may arise, resulting in a less values-consistent way of living. It is possible that for people in this non-clinical sample, the absence of committed action may be related to more passive behaviors, and consequently, less stress and/or anxiety. In contrast, withdrawing from VA is more likely to increase depression and is possibly reflected in the significant negative correlation between VA and depression (Cuijpers et al., 2007).

The results of this study suggest that ACT researchers and clinicians may consider using the CompACT-10 with U.S. populations, particularly where efficient screening and reduced participant burdens are priorities. Consistent with previous research, the 18- (Trindade et al., 2021; Tynan et al., 2022) and 15-item (Hsu et al., 2023) versions also demonstrated good psychometric properties and may be useful in research contexts requiring more comprehensive assessment of psychological flexibility, especially when detailed subscale interpretations are needed. All three short-form versions address shortcomings of the initial CompACT, including redundant items, factor imbalance (10 OE items, 8 VA items, and 5 BA items), and poor overall psychometrics. In additional to the statistically driven process, the retained statements on the 10-item version were selected for their clear wording and overall quality (Golijani-Moghaddam et al., 2023). Despite these strengths, as with any short-form measure, it is important to note that a potential consequence of fewer items is reduced conceptual coverage and threats to content validity.

This study has several limitations. First, CompACT-23 data were collected from a single sample, and all short-form versions were analyzed using the same data set. Testing each short-form version on different samples would have improved study rigor. Second, this was a largely homogeneous sample (primarily White Americans), which precludes generalizability of findings. Third, the sample was recruited through Qualtrics, which poses several limitations, including limited transparency regarding the composition and fluctuation of its respondent pool. These factors complicate efforts to assess sample representativeness and may introduce selection biases (Boas et al., 2020; Miller et al., 2020). As such, generalizability to broader U.S. populations should be interpreted with caution. Fourth, data from this study was assessed using a cross-sectional design. Additional research is needed to analyze the factor structure of the CompACT-10 in longitudinal clinical trials. Despite these limitations, the current results generally support the psychometrics of the CompACT-10 in community-dwelling, non-clinical adult samples.

## Supplementary Material

1

## Figures and Tables

**Table 1:** Goodness of Fit Statistics for 23–, 18-, 15-, and 10-Item Versions of the Comprehensive Assessment of ACT Processes Measure (CompACT).

	SBχ^2^	*df*	*p*	RMSEA	RMSEA 90 % CI	CFI	SRMR	BIC
23-item	393.39	69.33	< 0.001	0.09	0.08, 0.09	0.88	0.16	29885.31
18-item	173.33	54.98	< 0.001	0.07	0.06, 0.08	0.95	0.08	22606.67
15-item	147.63	42.98	< 0.001	0.07	0.06, 0.08	0.96	0.07	18431.69
10-item	81.96	20.70	< 0.001	0.07	0.06, 0.08	0.97	0.06	14179.43

*Note.* SBχ^2^ = Satorra-Bentler Adjusted Chi-Square; RMSEA = root mean square error of approximation; 90 % CI = 90 % confidence interval; CFI = comparative fit index; SRMR = standardized root mean square residual; BIC = Bayesian information criteria.

**Table 2:** Completely Standardized Confirmatory Factor Analysis Factor Loadings on the CompACT (n = 601).

CompACT item	23-item	18-item	15-item	10-item
1. I can identify the things that really matter to me in life and pursue them. (VA)	0.75	0.75	0.67	– -
2. One of my big goals is to be free from painful emotions. (R) (OE)	0.63	0.71	0.70	– -
3. I rush through meaningful activities without being really attentive to them. (R) (BA)	0.78	0.78	0.78	0.78
4. I try to stay busy to keep thoughts or feelings from coming. (R) (OE)	0.74	0.75	0.75	– -
5. I act in ways that are consistent with how I wish to live my life. (VA)	0.78	0.77	– -	0.74
6. I get so caught up in my thoughts that I am unable to do the things that I most want to do. (R) (OE)	0.76	– -	– -	– -
7. I make choices based on what is important to me, even if it is stressful. (VA)	0.69	0.69	0.68	– -
8. I tell myself that I shouldn’t have certain thoughts. (R) (OE)	0.75	0.72	0.72	0.71
9. I find it difficult to stay focused on what’s happening in the present. (R) (BA)	0.81	0.80	0.80	– -
10. I behave in line with my personal values. (VA)	0.85	0.85	0.80	0.87
11. I go out of my way to avoid situations that might bring difficult thoughts, feelings, or sensations. (R) (OE)	0.71	0.76	0.76	0.81
12. Even when doing the things that matter to me, I find myself doing them without paying attention. (R) (BA)	0.84	0.84	0.84	0.83
13. I am willing to fully experience whatever thoughts, feelings and sensations come up for me, without trying to change or defend against them. (OE)	−0.14	– -	– -	– -
14. I undertake things that are meaningful to me, even when I find it hard to do so. (VA)	0.71	0.71	– -	0.70
15. I work hard to keep out upsetting feelings. (R) (OE)	0.64	0.71	0.71	0.67
16. I do jobs or tasks automatically, without being aware of what I’m doing. (R) (BA)	0.77	0.78	0.78	– -
17. I am able to follow my long terms plans including times when progress is slow. (BA)	0.71	0.71	0.70	– -
18. Even when something is important to me, I’ll rarely do it if there is a chance it will upset me. (R) (OE)	0.75	– -	– -	– -
19. It seems I am “running on automatic” without much awareness of what I’m doing. (R) (BA)	0.86	0.86	0.86	0.85
20. Thoughts are just thoughts – they don’t control what I do. (OE)	−0.08	– -	– -	– -
21. My values are really reflected in my behavior. (BA)	0.80	0.80	– -	– -
22. I can take thoughts and feelings as they come, without attempting to control or avoid them. (OE)	−0.09	– -	– -	– -
23. I can keep going with something when it’s important to me. (VA)	0.78	0.78	0.83	0.77

*Note*: R = reverse-scored item. BA = Behavioral Awareness factor, OE = Openness to Experience factor, VA = Valued Action factor. In the 18-, 15-, and 10-item CompACT versions, items are presented in the same order as the 23-item version.

**Table 3:** Factor Correlations and Correlations between the 10-item Comprehensive Assessment of ACT Processes Measure (CompACT-10) and Other Study Variables.

	BA	OE	VA	SWLS	FFMQ-SF	SCS-SF	BRS	DASS-D	DASS-A	DASS-S	Gender
CompACT-10	– -	– -	– -	0.28**	0.71**	0.57**	0.55**	−0.55**	−0.47**	−0.48**	0.08
BA	– -	– -	0.18**	0.12**	0.63**	0.38**	0.41**	−0.52**	−0.47**	−0.49**	0.04
OE	0.55**	– -	– -	0.14**	0.50**	0.34**	0.33**	−0.43**	−0.42**	−0.41**	−0.01
VA	– -	0.19**	– -	0.30**	0.28**	0.41**	0.37**	−0.15**	−0.05	−0.06	0.03

*N* = 601. BA = Behavioral Awareness, OE = Openness to Experience, VA = Valued Action, SWLS = Satisfaction with Life Scale, FFMQ-SF = Five-Facet Mindfulness Questionnaire – Short Form, SCS-SF = Self-Compassion Scale – Short Form, BRS = Brief Resilience Scale, DASS-D = The Depression, Anxiety, and Stress Scale – Depression Subscale, DASS-A = The Depression, Anxiety, and Stress Scale – Anxiety Subscale, DASS-S = The Depression, Anxiety, and Stress Scale – Stress Subscale.

* *p* < 0.05

** *p* < 0.01.

## Data Availability

The dataset in the current study is available from the corresponding author upon request.

## Appendix A. Supplementary data

Supplementary data to this article can be found online at https://doi.org/10.1016/j.jbct.2025.100569.
