# Supplementary material for: Assessing the psychometric properties of the 10-item version of the comprehensive assessment of ACT processes (CompACT) in a community-based adult sample
Source: J Behav Cogn Ther. Author manuscript; Available in PMC 2026 Jul 9. (PMC13344365; doi:10.1016/j.jbct.2025.100569)
Supplement: 1 [file NIHMS2188372-supplement-1.docx]

**Supplemental Materials File**

Comprehensive Assessment of ACT Processes 10-Item Version (CompACT-10)

1. I rush through meaningful activities without being really attentive to them. (R) (BA)
2. I act in ways that are consistent with how I wish to live my life. (VA)
3. I tell myself that I shouldn’t have certain thoughts. (R) (OE)
4. I behave in line with my personal values. (VA)
5. I go out of my way to avoid situations that might bring difficult thoughts, feelings, or sensations. (R) (OE)
6. Even when doing the things that matter to me, I find myself doing them without paying attention. (R) (BA)
7. I undertake things that are meaningful to me, even when I find it hard to do so. (VA)
8. I work hard to keep out upsetting feelings. (R) (OE)
9. It seems I am “running on automatic” without much awareness of what I'm doing. (R) (BA)
10. I can keep going with something when it’s important to me. (VA)

*Note*. Items are scored on a 7-point Likert scale ranging from 0 = *strongly disagree* to 6 = *strongly agree*. R = reverse-scored item, BA = Behavioral Awareness factor, OE = Openness to Experience factor, VA = Valued Action factor.
